# Similarities of metabolomic disturbances in prematurity-associated obstructive lung disease to chronic obstructive pulmonary disease

**DOI:** 10.1038/s41598-024-73704-1

**Published:** 2024-10-07

**Authors:** Christopher W. Course, Philip A. Lewis, Sarah J. Kotecha, Michael Cousins, Kylie Hart, Kate J. Heesom, W. John Watkins, Sailesh Kotecha

**Affiliations:** 1 Department of Child Health, School of Medicine, Cardiff University, Heath Park, Cardiff, CF14 4XN UK; 2 Faculty of Life Sciences, University of Bristol, Bristol, UK; 3 Department of Paediatrics, Cardiff and Vale University Health Board, Cardiff, UK

**Keywords:** Prematurity, Spirometry, Metabolomics, Mass spectrometry, Fatty acids, Glutathione, Mechanisms of disease, Molecular medicine, Paediatrics, Neonatology

## Abstract

Prematurity-associated lung disease (PLD) is a long-term consequence of preterm-birth. Since the underlying mechanisms of PLD remain poorly characterised, we compared the urinary metabolome between recently described spirometry phenotypes of PLD. Preterm- and term-born children aged 7–12 years, from the Respiratory Health Outcomes in Neonates (RHiNO) cohort, underwent spirometry and urine collection. The urinary metabolome was analysed by gas chromatography time-of-flight mass spectrometry. Preterm-born children were classified into phenotypes of prematurity-associated obstructive lung disease (POLD, Forced expiratory volume in 1 s (FEV_1_) < lower limit of normal (LLN), FEV_1_/Forced Vital Capacity (FVC) < LLN), prematurity-associated preserved ratio impaired spirometry (pPRISm, FEV_1_ < LLN, FEV_1_/FVC ≥ LLN) and Preterm/Term controls (FEV_1_ ≥ LLN). Metabolite set enrichment analysis was used to link significantly altered metabolites between the groups with metabolic pathways. Univariable and multivariable linear regression models examined associations between early and current life factors and significantly altered metabolites of interest. Urine from 197 preterm- and 94 term-born children was analysed. 23 and 25 were classified into POLD and pPRISm groups respectively. Of 242 identified metabolites, 49 metabolites were significantly altered in the POLD group compared with Preterm controls. Decreased capric acid (log_2_ fold change − 0.23; *p* = 0.003), caprylic acid (− 0.18; 0.003) and ceratinic acid (− 0.64; 0.014) in the POLD group, when compared to preterm controls, were linked with reduced β-oxidation of very long chain fatty acids (*p* = 0.004). Reduced alanine (log_2_ fold change − 0.21; *p* = 0.046), glutamic acid (− 0.24; 0.023), and pyroglutamic acid (− 0.17; 0.035) were linked with decreased glutathione metabolism (*p* = 0.008). These metabolites remained significantly associated with POLD in multivariable models adjusting for early/current life factors. The pPRISm urinary metabolome was minimally changed when compared with preterm-born controls. When compared to term-born subjects, alterations in tryptophan metabolism were implicated (*p* = 0.01). The urinary metabolome in POLD showed significantly altered β-oxidation of fatty acids and glutathione metabolism, implying alterations in cellular metabolism and oxidative stress. Similar findings have been noted in adults with chronic obstructive pulmonary disease. Given the similarity of findings between the POLD group and those reported for COPD, the POLD group should be considered at future risk of developing COPD.

**Supplementary Information:**

The online version contains supplementary material available at 10.1038/s41598-024-73704-1.

## Introduction

Lung function impairments are known long-term consequences of preterm birth, including those with and without a neonatal diagnosis of bronchopulmonary dysplasia (BPD), also known as chronic lung disease of prematurity (CLD)^1–3^. Historically, studies have focused on pulmonary outcomes for those with BPD, however, increasingly immature gestational age at birth and intra-uterine growth restriction (IUGR) appear to be more strongly associated with prematurity-associated lung disease (PLD) in childhood^4^ for those who experienced a contemporary standard of neonatal care. We recently described multiple phenotypes of PLD, including prematurity-associated obstructive lung disease (POLD) and prematurity-associated preserved ratio impaired spirometry (pPRISm) spirometry patterns^5^. These were differentially associated with early- and current-life factors, with POLD being significantly associated with a neonatal history of IUGR and BPD, in contrast to pPRISm which was associated with a lower current body mass index. There is concern that PLD increases the risk of early-onset chronic obstructive pulmonary disease (COPD) in adult life^2,6^.

Whilst a proportion of individuals with PLD will respond to inhaled therapies^7^, the biological pathways implicated in the development of these PLD-associated phenotypes remain unclear, with most previous mechanistic work focusing on BPD diagnosed in the neonatal period^8^ rather than current spirometry deficits. This limits the ability to accurately identify PLD endotypes and target potential treatments. In addition to being a readily accessible biofluid, urine lacks the same homeostatic mechanisms as blood, therefore systemic metabolite changes accumulate and the urinary metabolome may show alterations prior to symptoms or histopathological changes, reflecting an earlier stage of pathogenesis^9^. Therefore, whilst not a lung-specific sample, urine has been extensively used in metabolomic studies of respiratory disease, such as asthma and COPD, where it has been demonstrated to show metabolomic alterations before they occur in serum^10^. Alterations in glutathione metabolism, lipid metabolism and lipid peroxidation have been implicated in severe asthma phenotypes and COPD^11^. The early urinary metabolome in preterm infants demonstrates specific changes, including increased myoinositol and taurine, that predict later development of BPD^12^, whilst the exhaled breath condensate from adolescent BPD survivors demonstrates distinct metabolite abnormalities possibly related to pulmonary surfactant composition and anti-inflammatory pathways^13^. However, how these patterns relate to current lung function of preterm-born children remains unclear.

We have previously reported on the urinary proteome of children with PLD, finding associations with T-lymphocyte biology in pPRISm, in contrast to POLD where neutrophil and macrophage activity appeared to be altered^14^. However, the urinary metabolome of children with PLD has yet to be studied. We, therefore, performed an exploratory metabolomic analysis of urine taken from preterm-born, school-aged children with term-born matched controls, aiming to delineate the metabolic pathways underlying the PLD phenotypes of POLD and pPRISm.

## Methods

### Participants

This study was conducted on children recruited to the Respiratory Health Outcomes in Neonates study (RHiNO, EudraCT: 2015-003712-20) which has been described previously^4,7,14^. Briefly, suitable candidates from our previous cohort study^15^ were supplemented with additional preterm-born children identified by the NHS Wales Informatics Service and were invited to join the RHiNO study via a mailed respiratory and neurodevelopmental questionnaire, if they were born ≤ 34 or ≥ 37 week’s gestation and were aged 7–12 years old. Prospective recruitment occurred between November 2016 and September 2019. Children with significant congenital malformations, cardiopulmonary or neuromuscular disease were excluded. Ethical approval was obtained from the South-West Bristol Research Ethics Committee (15/SW/0289). Parents gave informed written consent and children provided assent. The study was conducted according to the Good Clinical Practice (GCP) guidelines and the Declaration of Helsinki.

### Lung function assessment

Spirometry (Microloop, Care Fusion, UK) was performed by trained research nurses to American Thoracic Society (ATS)/European Respiratory Society (ERS) guidelines^16^ and normalised using Global Lung Initiative (GLI) references^17^. Respiratory medications were withheld prior to assessment (short- and long-acting β2-agonists for 8- and 48-h respectively; inhaled corticosteroids for 24 h; leukotriene receptor antagonists for 48 h) and children were free of respiratory infections for at least 3 weeks prior to testing. Low lung function in preterm-born children (PLD) was defined as FEV_1_below the lower limit of normal (LLN). Those with PLD were further categorised, as previously described^5^, into POLD (FEV_1_ < LLN and FEV_1_/FVC < LLN) and pPRISm (FEV_1_ < LLN and FEV_1_/FVC ≥ LLN) groups. Preterm-born control (PT_c_) and Term-born children had FEV_1_≥ LLN. BPD was defined as oxygen-dependency for 28 days of age or greater for those born < 32 weeks’ gestation and at 56 days of age for those born ≥ 32 weeks’ gestation)^18^. IUGR was defined as birthweight < 10th percentile adjusted for sex and gestation (LMS Growth version 2.77, Medical Research Council, UK). Neonatal history was corroborated with medical records. Doctor-diagnosed asthma and history of wheeze was self-reported by parents.

### Urine sampling

Urine samples were obtained at the time of spirometry, and immediately placed on ice. Samples were then aliquoted and stored at − 80 °C as soon as possible on the day of collection until further processing and analysis.

### Metabolome analysis

Urine samples were analysed using Gas Chromatography Time-of-Flight Mass Spectrometry (GCTOF-MS) at the West Coast Metabolomics Centre (University of California, Davis)^19^, who have previously published their analytical method^19^ and which is described in detail in the supplementary methods (Supplementary File). The analytical method was aimed at identifying constituents of primary metabolism (carbohydrates and sugar phosphates, amino acids, hydroxyl acids, free fatty acids, purines, pyrimidines, aromatics, and exposome-derived chemicals). Quantification of metabolites are reported as spectral peak height of the unique ion detected (m/z value) at the specific retention index. The urine samples were analysed across 33 batches, with a pool sample analysed with each batch to control for between-batch normalisation.

### Statistical analysis

Demographics were compared using chi-squared or one-way ANOVA with Bonferroni correction as appropriate. Metabolite quantities were normalised using creatinine (as detected by MS), as recommended to account for dilutional effects^20^, log_10_ transformed and visually inspected for normality. Urine creatinine values for the four study groups are shown in Supplementary File Fig. 1. Metabolites with mean and median peak intensities below the limit of detection were removed to ensure robust statistical comparisons. Fold changes between study and control groups were calculated using mean metabolite quantity for each group. Metabolite Set Enrichment Analysis (MSEA; identifying metabolic processes linked to significantly altered metabolites) was performed on all significantly altered metabolites between groups using the Small Molecule Pathways Database (SMPDB) (https://www.smpdb.ca), which is based upon the Human Metabolome Database (HMDB). Biologically important metabolites identified through MSEA were then compared between the four study groups using ANOVA with post-hoc Bonferroni correction to understand the metabolites relationships between the two PLD groups, namely POLD and pPRISm, as well as both the preterm- and term-born control groups. Univariable linear regression models identified significant associations between participant characteristics, spirometry values and metabolites of interest within the preterm-born cohort. Univariable associations with a p-value < 0.1 were combined into multivariable linear regression models to determine overall contributions to any significant differences observed. Owing to the exploratory nature of the analyses, *p* < 0.05 was considered statistically significant. Analyses were performed using R v4.0.4 (R Foundation for Statistical Computing, Austria) and MetaboAnalyst v5.0 (www.metaboanalyst.ca).

## Results

From 768 children (565 Preterm-born and 203 Term-born) recruited to RHiNO, urine was analysed from 292 participants; 1 sample from a PT_c_ subject was excluded as an outlier due to minimal overall metabolite detection. Demographics of the remaining 291 participants are given in Table 1. Preterm-born children had higher rates of doctor-diagnosed asthma than the Term-born group (41 (20.8%) vs. 6 (6.4%), p ≤ 0.002). 51 (25.9%) of the Preterm-born subjects had BPD diagnosed in infancy (21 [41.2%] mild, 30 [58.8%] moderate/severe^18^), and 48 (24.4%) had an FEV_1_ < LLN. Of those, 25 (52.1%) were classified as pPRISm and 23 (47.9%) as POLD. 242 metabolites were detected and annotated in total, with 238 (98.4%) metabolites having mean and median abundances above the limit of detection across all samples (Supplementary File Table 1).

**Table 1:** Participant demographics.

Variable	Term born (≥ 37/40) n = 94	Preterm born (≤ 34/40) n = 197	Preterm born Controls n = 149	POLD n = 23	pPRISm n = 25
**Current characteristics**					
Sex (male), n (%)	50 (53.2)	108 (54.8)	83 (56.1)	9 (39.1)	16 (64.0)
Ethnicity (white), n (%)	91 (96.8)	190 (96.4)	143 (96.0)	22 (95.7)	25 (100)
Age at testing (years), mean (SD)	9.7 (1.2)	10.0 (1.2)	10.0 (1.2)	9.9 (1.4)	10.2 (1.2)
Weight (kg), mean (SD)	37.1 (10.8)	36.6 (10.3)	37.1 (10.1)	33.5 (10.8)	36.7 (10.7)
Body mass index (kg/m^2^), mean (SD)	18.0 (3.4)	17.9 (3.3)	18.1 (3.2)	16.8 (3.1)	17.4 (3.5)
Wheeze-ever, n (%)	25 (26.6)	108 (54.8)***	76 (51.0)	19 (82.6)^††$^	13 (52.0)
Doctor-diagnosed asthma, n (%)	6 (6.4)	41 (20.8)**	26 (17.5)	9 (39.1)^†^	6 (24.0)
Short-acting β_2_ agonist use, n (%)	4 (4.3)	33 (16.8)**	20 (13.4)	9 (39.1)^††^	4 (16.0)
Long-acting β_2_ agonist use, n (%)	1 (1.1)	5 (2.5)	3 (2.0)	1 (4.3)	1 (4.0)
Inhaled corticosteroid use, n (%)	4 (4.3)	26 (13.2)*	15 (10.1)	8 (34.8)^††^	3 (12.0)
Leukotriene receptor antagonists use, n (%)	1 (1.1)	3 (1.5)	2 (1.3)	1 (4.3)	0 (0)
**Neonatal characteristics**					
Gestational age (weeks), mean (SD)	39.9 (1.2)	30.7 (2.8)***	30.9 (2.8)	29.5 (2.4)	30.7 (3.1)
Birthweight (g), mean (SD)	3522 (522)	1666 (607)***	1731 (590)	1313 (578)^††^	1605 (639)
Birthweight (z-score), mean (SD)	0.12 (1.0)	0.20 (1.35)	0.33 (1.31)	− 0.35 (1.56)	− 0.12 (1.22)
Intrauterine growth restriction, n (%)	4 (4.3)	29 (14.7)**	18 (12.1)	9 (39.1)^†††$^	2 (8.0)
Antenatal smoking, n (%)	4 (4.3)	23 (11.7)^‡^*	19 (12.8)	2 (8.7)	2 (9.1)
Antenatal steroids, n (%)	2 (2.1)	167 (89.8)^‡^***	127 (85.2)	18 (78.3)	22 (88.0)
Mechanical ventilation, n (%)	1 (1.1)	82 (41.6)***	61 (40.9)	14 (60.9)	7 (28.0)
Bronchopulmonary dysplasia (BPD), n (%)	0 (0)	51 (25.9)***	33 (22.2)	11 (47.8)^††^	7 (28.0)

Preterm born vs. Term born: *p < 0.05, **p < 0.01, ***p < 0.001.

pPRISm or POLD vs. Preterm born control: ^†^p < 0.05, ^††^p < 0.01, ^†††^p < 0.001.

pPRISm vs.POLD: ^$^p < 0.05, ^$$^p < 0.01, ^$$$^p < 0.001.

All between-group comparisons Chi-squared/independent samples t-test/ANOVA with post-hoc Bonferroni correction as appropriate.

*pPRISm* prematurity-related preserved ratio with impaired spirometry, *POLD* prematurity-related obstructive lung disease.

^‡^Antenatal steroid data missing for 11 preterm-born children. Antenatal smoking data missing for 4 preterm-born children.

### Comparisons between POLD and preterm- and term-control groups

Comparison between the POLD group and PT_c_ group revealed several significant differences (Table 1) including increased wheeze-ever (82.6% vs. 51.0%, *p* = 0.009), doctor-diagnosed asthma (39.1% vs. 17.5%, *p* = 0.017), IUGR (39.1% vs. 12.1%, p = < 0.001) and BPD (47.8% vs. 22.2%, *p* = 0.009). The POLD group also had a higher use of short-acting β_2_ agonists and inhaled corticosteroids compared to PTc. When compared to the pPRISm group (Table 1), POLD had higher wheeze-ever (82.6% vs. 52.0%, *p* = 0.025) and higher rates of IUGR (39.1% vs. 8.0%, *p* = 0.01).

**Fig. 1:**
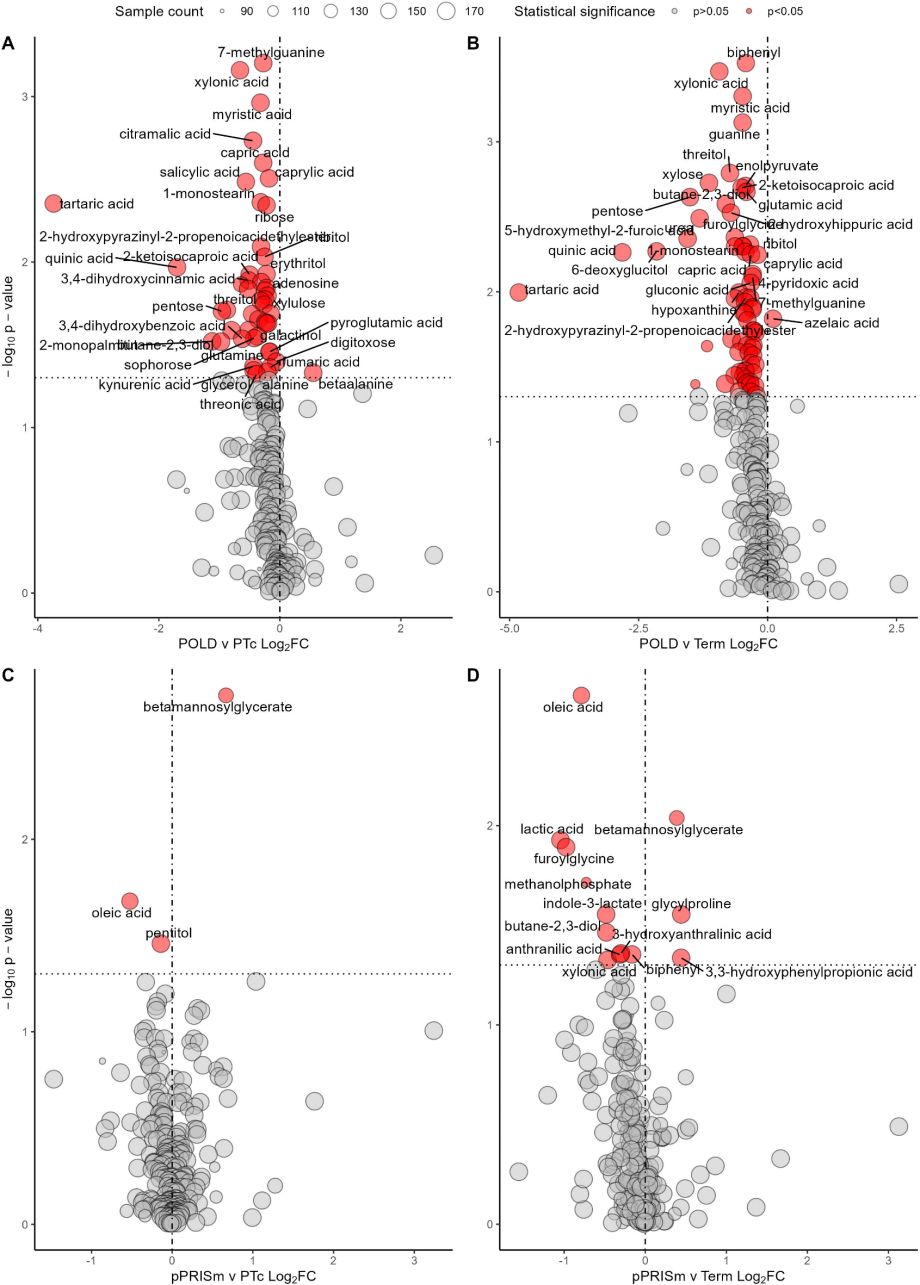
Volcano plots demonstrating significantly altered metabolites between groups (**A**) POLD vs. PT_c_ (**B**) POLD vs. Term (**C**) pPRISm vs. PT_c_ (**D**) pPRISm vs. Term. Fold changes between groups log_2_ transformed for visualization. Vertical line represents a Log_2_FC of 0. Horizontal line is equivalent to p-value 0.05. Size of point is relative to number of samples in which metabolite was detected. Metabolite name given if p < 0.05. *Log*_*2*_*FC* Log_2_ fold-change between groups.

Of 238 detected metabolites detected in samples from the POLD group, 204 were present in every sample analysed. 49 (20.6%) of these metabolites were significantly altered when compared to PT_c_ (Fig. 1; Table 2), and 69 (29.0%) when compared to Term-born children (Fig. 1, Supplementary File Table 2), with 31 metabolites common between the two comparisons. Interestingly, all significantly altered metabolites were of lower quantity in the POLD group when compared with the PT_c_ group, apart from beta-alanine, which was elevated (log_2_FC 0.55, *p* = 0.047).

**Table 2:** Significantly altered metabolites in POLD and pPRISm groups when compared to preterm-born controls.

Metabolite	Retention index	m/z	PubChem ID	% of samples	Log_2_FC	p-value
POLD vs. PT_c_ n = 23 v 149						
7-Methylguanine	768706	294	11361	100	− 0.27	< 0.001
Xylonic acid	589278	333	6602431	99.4	− 0.66	< 0.001
Myristic acid	634414	285	11005	100	− 0.32	0.001
Citramalic acid	456203	247	1081	100	− 0.44	0.002
Capric acid	452386	229	2969	100	− 0.28	0.003
Caprylic acid	343457	201	379	100	− 0.18	0.003
Salicylic acid	480699	267	338	98.8	− 0.56	0.003
1-Monostearin	959214	203	24699	100	− 0.32	0.004
Tartaric acid	534291	292	444305	99.4	− 3.74	0.004
Ribose	553071	217	10975657	100	− 0.22	0.005
2-Hydroxypyrazinyl-2-propenoicacidethylester	493127	121	5371086	100	− 0.31	0.008
Ribitol	575497	217	827	100	− 0.25	0.009
Quinic acid	634900	345	6508	100	− 1.70	0.011
Erythritol	471922	217	222285	100	− 0.22	0.012
2-Ketoisocaproic acid	290473	89	70	100	− 0.50	0.012
34-Dihydroxycinnamic acid	748847	219	689043	100	− 0.52	0.013
28-Dihydroxyquinoline	626989	290	97250	100	− 0.31	0.013
Ceratinic acid	1033286	145	10469	86.0	− 0.64	0.014
Adenosine	918039	236	60961	100	− 0.22	0.014
UDP-glucuronic acid	585473	217	17473	100	− 0.52	0.015
Benzoic acid	339067	179	243	100	− 0.24	0.015
Xylitol	567437	217	6912	100	− 0.20	0.016
Xylulose	553450	173	439205	100	− 0.24	0.017
Threitol	467595	217	169019	100	− 0.32	0.017
Biphenyl	426625	154	7095	97.1	− 0.26	0.018
2-Hydroxyhippuric acid	725465	206	10253	100	− 0.88	0.019
Pentose	540818	103	229	100	− 0.96	0.020
Isothreonic acid	489385	292	151152	100	− 0.15	0.021
Lactose	929908	204	11333	98.3	− 0.46	0.021
*N*-Acetylmannosamine	722897	319	439281	100	− 0.35	0.022
Glutamic acid	529100	246	33032	100	− 0.24	0.023
Glucuronic acid	665901	333	94715	100	− 0.20	0.023
Xanthine	701688	353	1188	100	− 0.22	0.024
2-Picolinic acid	383668	180	1018	100	− 0.52	0.026
Xylose	544100	103	135191	100	− 0.80	0.026
Galactinol	1015529	204	N/A	100	− 0.37	0.028
Sophorose	959716	319	N/A	98.3	− 0.42	0.029
34-Dihydroxybenzoic acid	620200	193	72	100	− 0.62	0.029
2-Monopalmitin	890356	129	123409	100	− 1.12	0.030
Butane-23-diol	205778	117	262	100	− 0.97	0.030
Pyroglutamic acid	485935	156	7405	100	− 0.17	0.035
Glutamine	600000	156	5961	100	− 0.17	0.035
Digitoxose	521798	117	94168	100	− 0.06	0.040
Kynurenic acid	726186	231	3845	100	− 0.44	0.043
Fumaric acid	390016	245	444972	100	− 0.16	0.043
Glycerol	344466	205	753	100	− 0.43	0.045
Alanine	244189	116	5950	100	− 0.21	0.046
Beta-alanine	435564	248	239	100	0.55	0.047
Threonic acid	497572	292	5460407	100	− 0.39	0.048
pPRISm vs. PT_c_ n = 25 v 149						
Beta-mannosyl glycerate	774364	204	5460194	81.6	0.67	0.002
Oleic acid	781527	339	445639	91.4	− 0.52	0.021
Pentitol	563801	307	827	100	− 0.14	0.035

*m/z* mass-to-charge ratio, *Log*_*2*_*FC* Log_2_ fold change between groups.

**Fig. 2:**
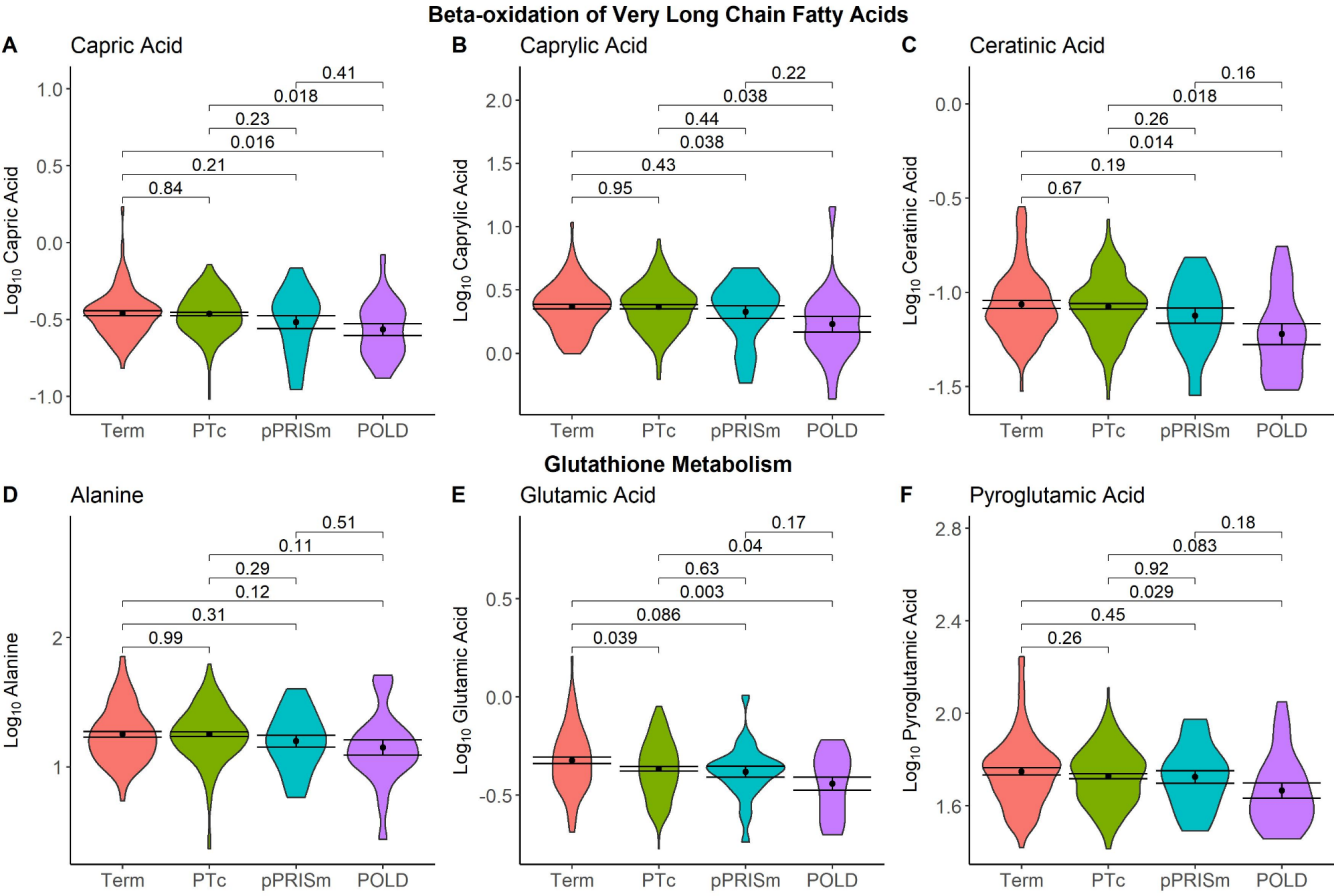
Violin plots of significantly altered metabolites in POLD group, grouped by associated metabolic process. Black dot and bars show mean and standard error of the mean (SEM). Bars give *p* values from ANOVA with post-hoc Bonferroni correction for between group comparisons.

**Fig. 3:**
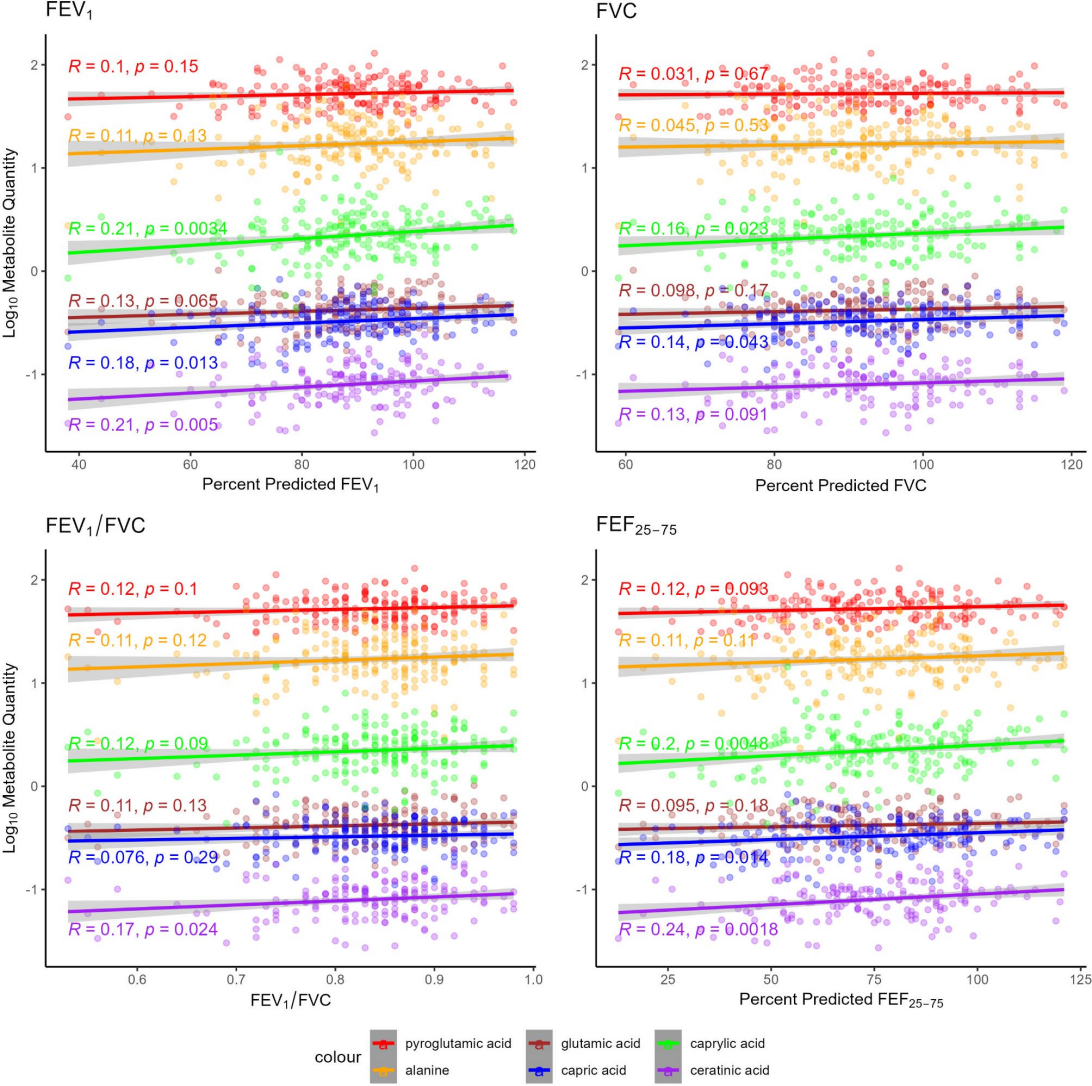
Scatter plots with linear regression lines demonstrating relationship between metabolites of interest and spirometry variables in preterm-born children. Points represent individual metabolite measurements. Line represents linear regression model, with 95% confidence interval represented by grey shading. *FEV*_*1*_ forced expiratory volume in one second, *FVC* forced vital capacity, *FEF*_*25–75*_ forced expiratory flow between 25 and 75% of vital capacity.

MSEA mapped 14 significantly altered metabolites between the POLD and PT_c_ groups to nine significantly altered metabolic processes (Table 3). Capric acid (log_2_FC − 0.28, *p* = 0.003), caprylic acid (− 0.18, 0.003), and ceratinic acid (− 0.64, 0.014) were linked with β-oxidation of very-long chain fatty acids (*p* = 0.004). Alanine (log_2_FC − 0.21, *p* = 0.046), glutamic acid (− 0.24, 0.023) and pyroglutamic acid (− 0.17, 0.035) were linked with glutathione metabolism (*p* = 0.008) (Supplementary File Fig. 2). Comparisons of these six metabolites, all of which were mapped by MSEA to metabolic processes with high enrichment ratios (β-oxidation of very-long chain fatty acids ER 8.6, glutathione metabolism ER 7.0) and high significance values (*p* < 0.01), between all four study groups are shown in Fig. 2. Significant reductions in capric acid, caprylic acid, ceratinic acid and glutamic acid were observed when compared to both the PT_c_ and Term-born groups (*p* < 0.05). In multiple group comparisons, pyroglutamic acid was significantly lower in the POLD group when compared to the Term-born group (*p* = 0.029), and near significantly lower when compared to PT_c_ (*p* = 0.083). Univariable and multivariable linear regression analyses of these six metabolites with early- and current-life factors in the preterm-born group are given in Table 4. Alanine and glutamic acid had a significant association with only POLD in univariable analysis (*p* = 0.046 and 0.022 respectively). In multivariable analysis, capric acid, caprylic acid, ceratinic acid and pyroglutamic acid all maintained a significant association with the POLD group (*p* = 0.009, 0.025, 0.035 and 0.046 respectively). Figure 3 shows the relationship between these six metabolites and spirometry values (percent predicted forced expiratory volume in one second (FEV_1_), percent predicted forced vital capacity (FVC), FEV_1_/FVC and percent predicted forced expiratory flow between 25 and 75% of vital capacity (FEF_25−75%_)). Significant associations were seen between FEV_1_ and capric acid, caprylic acid and ceratininc acid (*p* = 0.013, 0.0034 and 0.005 respectively), FVC and capric and caprylic acid (*p* = 0.043 and 0.028 respectively), FEV_1_/FVC and ceratinic acid (*p* = 0.024), and FEF_25−75_ and capric acid, caprylic and ceratinic acid (*p* = 0.014, 0.0048 and 0.0018 respectively).

**Table 3:** Metabolite set enrichment analysis demonstrating altered biological processes implicated by significantly altered metabolite quantities between POLD and pPRISm groups when compared to preterm- and term-born controls.

Metabolic process	Significantly altered metabolites	Enrichment ratio	p-value
POLD vs. PT_c_			
Urea cycle	Alanine, fumaric acid, glutamic acid, glutamine	6.7	0.002
β-oxidation of very long chain fatty acids	Capric acid, caprylic acid, ceratinic acid	8.6	0.004
Aspartate metabolism	Beta-alanine, fumaric acid, glutamic acid, glutamine	5.6	0.005
Glutathione metabolism	Alanine, glutamic acid, pyroglutamic acid	7.0	0.008
Purine metabolism	Adenosine, fumaric acid, glutamic acid, glutamine, xanthine	3.3	0.014
Glucose-alanine cycle	Alanine, glutamic acid	7.4	0.027
Amino sugar metabolism	Glutamic acid, glutamine, N-acetylmannosamine	4.4	0.027
Fatty acid biosynthesis	Capric acid, caprylic acid, myristic acid	4.2	0.032
Alanine metabolism	Alanine, glutamic acid	6.0	0.045
POLD vs. term			
Aspartate metabolism	Asparagine, glutamic acid, N-acetyl-L-aspartic acid, pyrophosphate, ureidosuccinic acid	4.4	0.004
Galactose metabolism	D-mannose, galactinol, glycerol, pyrophosphate, raffinose,	4.1	0.006
Purine metabolism	Adenine, guanine, glutamic acid, hypoxanthine, pyrophosphate, uric acid, xanthine	2.9	0.007
pPRISm vs. PT_c_			
No significant enrichment			
pPRISm vs. term			
Tryptophan metabolism	3-hydroxyanthranilic acid, anthranilic acid	11.4	0.01

*POLD* prematurity-associated obstructive lung disease, *pPRISm* prematurity-associated preserved ratio impaired spirometry, *PT*_*c*_ preterm-born controls.

**Table 4:** Univariable and multivariable linear regression analyses of identified metabolites of interest with early and current life factors in preterm-born children.

Variable	β-oxidation of very long chain fatty acids									Glutathione metabolism								
	Capric acid			Caprylic acid			Ceratinic acid			Alanine			Glutamic acid			Pyroglutamic acid		
	Beta	SE	p	Beta	SE	p	Beta	SE	p	Beta	SE	p	Beta	SE	p	Beta	SE	p
Univariable modelsh																		
Sex, ref = Male	− 0.05	0.02	0.019*	− 0.02	0.03	0.51	− 0.04	0.03	0.17	− 0.04	0.03	0.19	0.03	0.02	0.15	− 0.06	0.02	< 0.001*
Age at testing, years	0.01	0.01	0.22	0.01	0.01	0.62	0.01	0.01	0.27	0.02	0.01	0.21	0.01	0.01	0.25	− 0.01	0.01	0.11
Weight, z-score	0.01	0.01	0.60	− 0.001	0.01	0.94	0.003	0.01	0.82	− 0.01	0.01	0.55	0.01	0.01	0.34	− 0.02	0.01	0.047*
BMI, z-score	0.002	0.01	0.81	− 0.01	0.01	0.59	< 0.001	0.01	0.93	− 0.02	0.01	0.19	0.01	0.01	0.44	− 0.02	0.01	0.024*
Gestational age, weeks	0.003	0.004	0.41	0.01	0.01	0.04*	0.005	0.005	0.33	0.01	0.01	0.35	− 0.003	0.004	0.46	< 0.001	0.004	0.99
Birthweight, z-score	− 0.01	0.01	0.18	− 0.01	0.01	0.36	− 0.01	0.01	0.28	− 0.002	0.01	0.83	− 0.01	0.01	0.11	− 0.01	0.01	*0.09*
IUGR, ref = No IUGR	− 0.01	0.03	0.70	− 0.04	0.04	0.35	− 0.004	0.04	0.92	− 0.05	0.05	0.31	0.01	0.03	0.71	0.01	0.03	0.84
BPD, ref = No BPD	0.01	0.03	0.66	0.01	0.04	0.89	− 0.003	0.03	0.92	0.01	0.04	0.89	0.03	0.02	0.24	0.03	0.02	0.12
POLD, ref = PT_c_	− 0.10	0.04	0.004*	− 0.14	0.05	0.004*	− 0.15	0.05	0.001*	− 0.10	0.05	0.046*	− 0.08	0.03	0.022*	− 0.06	0.03	0.036*
pPRISm, ref = PT_c_	− 0.05	0.03	0.12	− 0.04	0.05	0.37	− 0.05	0.04	0.22	− 0.05	0.05	0.29	− 0.02	0.03	0.64	− 0.003	0.03	0.91
Asthma, ref = No	− 0.04	0.03	0.20	− 0.09	0.04	0.021*	− 0.10	0.03	0.002*	− 0.05	0.04	0.18	− 0.03	0.03	0.25	− 0.07	0.02	0.003*
SABA, ref = no	− 0.02	0.03	0.63	− 0.04	0.04	0.33	− 0.08	0.04	0.022*	− 0.02	0.04	0.61	− 0.01	0.03	0.73	− 0.03	0.03	0.22
ICS, ref = no	− 0.04	0.03	0.20	− 0.09	0.05	0.06	− 0.11	0.04	0.003*	− 0.07	0.05	0.13	− 0.02	0.03	0.55	− 0.06	0.03	0.035*
Multivariable models																		
Sex, ref = Male	− 0.05	0.02	0.03*	–	–	–	**–**	**–**	**–**	Not taken forward for multivariable analysis			Not taken forward for multivariable analysis			0.07	0.02	< 0.001*
BMI, z-score	**–**	**–**	**–**	**–**	**–**	**–**	**–**	**–**	**–**							− 0.02	0.01	0.039*
Gestational age, weeks	**–**	**–**	**–**	0.01	0.005	0.10	**–**	**–**	**–**							**–**	**–**	**–**
Birthweight, z**-**score	**–**	**–**	**–**	**–**	**–**	**–**	**–**	**–**	**–**							− 0.02	0.01	0.006*
POLD, ref = PT_c_	− 0.09	0.04	0.009*	− 0.06	0.05	0.025*	− 0.10	0.05	0.035*							− 0.06	0.03	0.046*
Asthma, ref = No	–	–	–	− 0.06	0.04	0.06	− 0.06	0.04	0.14							0.04	0.03	0.21
SABA, ref = no	–	–	–	–	–	–	0.003	0.06	0.96							–	–	–
ICS, ref = no	–	–	–	–	–	–	− 0.08	0.06	0.19							− 0.04	0.03	0.29

Variables with a p < 0.1 in univariable analysis combined into multivariable model.

*SE* standard error, *BMI* body mass index, *IUGR* intrauterine growth restriction, *POLD* prematurity-associated obstructive lung disease, *SABA* short-acting β_2_ agonist, ICS: inhaled corticosteroids.

*and bold indicates p < 0.05. Dashes indicate a variable where p ≥ 0.1 in univariable analysis and therefore not included in multivariable model.

In addition to alanine and glutamic acid, significant differences in fumaric acid (log_2_FC − 0.16, *p* = 0.043) and glutamine (− 0.17, 0.035) were linked by MSEA to urea cycle metabolism (*p* = 0.002) (Table 3). In addition to fumaric acid, glutamic acid, and glutamine, MSEA linked a significant increase of beta-alanine (log_2_FC 0.55, *p* = 0.047) to aspartate metabolism (*p* = 0.005). Results of linear regression analyses for these metabolites are shown in Supplementary File Table 2. Fumaric acid and glutamine remained significantly associated with the POLD group in multivariable models (*p* = 0.021 and 0.012 respectively), but beta-alanine was no longer significant on multivariable analysis (*p* = 0.16).

**Fig. 4:**
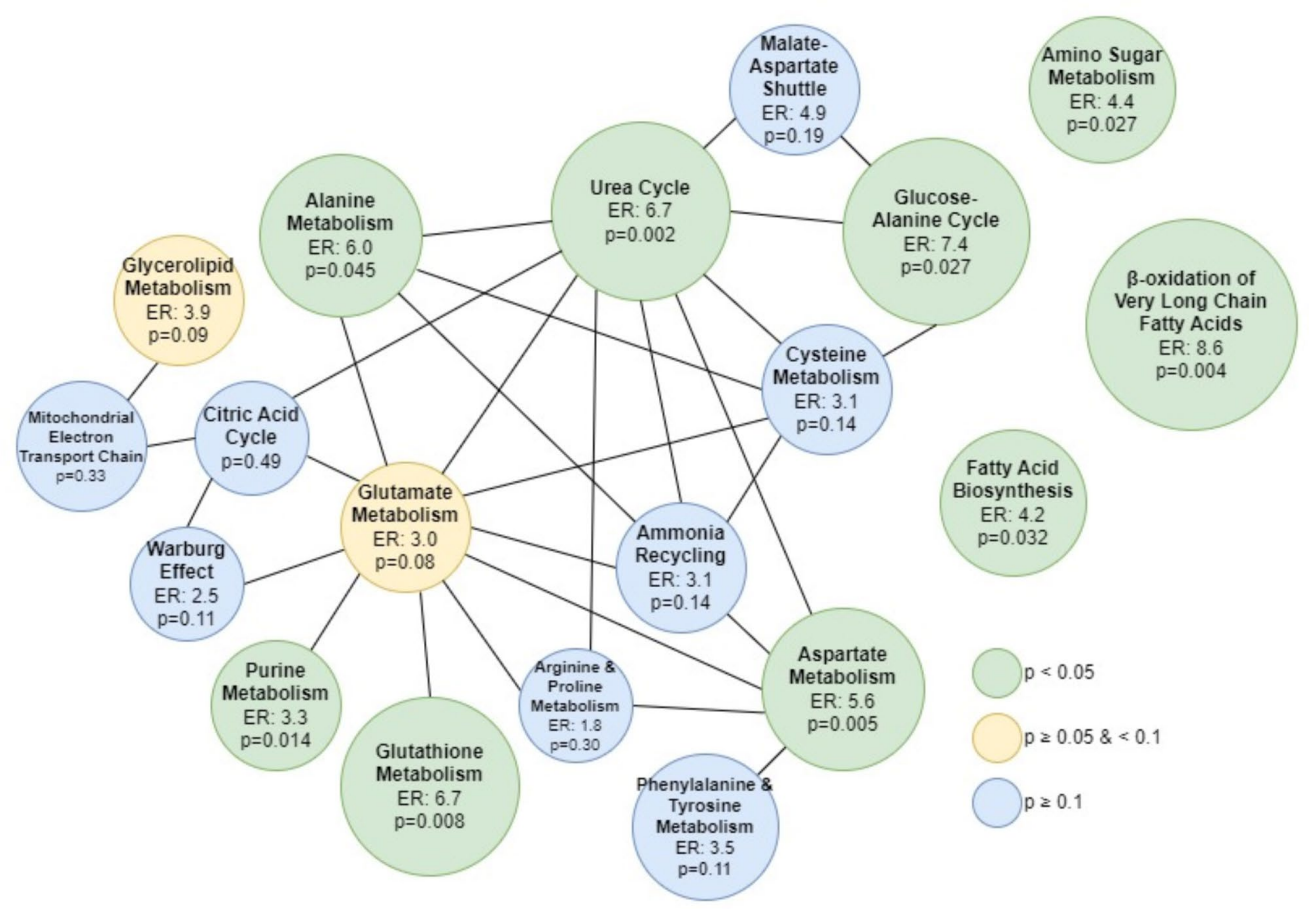
Interactions between metabolic processes identified by Metabolite Set Enrichment Analysis (MSEA) module in Metaboanalyst v5 as significantly enriched in POLD group compared to PT_c_. Processes colour coded according to their p-value. Size of circle relative to enrichment ratio of metabolic process. Two metabolic processes are connected by an edge if they share more than 25% of their respective metabolites. *ER* Enrichment ratio.

Figure 4 shows links between the significantly altered metabolic processes between the POLD and PT_c_ groups identified by MSEA, where linked processes are defined by sharing > 25% of their metabolites. Direct relationships exist between alanine metabolism, aspartate metabolism and urea cycle. Purine and glutathione metabolism were also linked by glutamate metabolism, which showed a near-significant enrichment (*p* = 0.08).

We detected 69 metabolites which were significantly altered in the POLD group when compared to the Term-group (Supplementary File Table 2). MSEA linked 14 to three significantly altered metabolic processes (Table 3). As with the comparison with the PT_c_ group, aspartate metabolism (*p* = 0.004) and purine metabolism (*p* = 0.007) showed significant enrichment, however glutamic acid was the only common metabolite observed. Galactose metabolism showed a significant enrichment on comparison of the POLD with the term-born group (*p* = 0.006) (Table 3).

### Comparisons between pprism and preterm- and term-control groups

Of 238 detected metabolites detected in samples from the pPRISm group, 204 were present in every sample analysed. 3 (1.3%) of these metabolites were significantly altered when compared to PT_c_ (Fig. 1; Table 2), and 13 (5.5%) when compared to the Term-born subjects (Fig. 1, Supplementary File Table 2), with two metabolites being common between the two comparisons (beta-mannosyl glycerate and oleic acid).

Beta-mannosyl glycerate (log_2_FC 0.67, *p* = 0.002), oleic acid (− 0.52, 0.021) and pentitol (− 0.14, 0.035) were significantly altered in the pPRISm group when compared to PT_c_, however MSEA showed no significant associations with any specific metabolic process (Table 3). Two altered metabolites in the pPRISm group when compared to the Term-born group (3-hydroxyanthranilic acid [log_2_FC − 0.30, 0.044] and anthranilic acid [− 0.31, 0.044]) were significantly mapped by MSEA to tryptophan metabolism (*p* = 0.01).

## Discussion

In this novel, exploratory metabolomic analysis of urine from school-aged children with PLD, we demonstrate significant differences in multiple metabolites linked with several metabolic processes in the POLD group when compared to preterm- and term-born controls. Of particular interest were significant decreases in metabolites consumed and produced during fatty acid biosynthesis and metabolism, especially β-oxidation of very-long chain fatty acids, and glutathione metabolism, findings which are similar to those reported in studies of adults with COPD^10,21–23^. We have previously demonstrated that a neonatal history of BPD is significantly associated with development of an obstructive spirometry pattern in childhood^5^, and our recently published meta-analysis has demonstrated that this airway obstruction likely increases over the life course^2^. There is increasing concern that PLD predisposes to early-onset COPD in adulthood^6^. Our current exploratory metabolomic analyses suggest that the altered metabolic activity present in childhood for those with a POLD phenotype is similar to adult studies of COPD, even after adjusting for relevant early- and current-life factors in regression modelling. In contrast, minimal differences were noted for the urinary metabolome in the pPRISm phenotype when compared with the preterm- and term-born controls, implying less systemic active metabolic processes occurring in this group.

β-oxidation of very-long chain fatty acids occur in perioxisomes, where fatty acids are broken down before transportation to mitochondria, where further fatty acid degradation and energy release occurs^24^. The increased energy requirements secondary to airway inflammation and increased work of breathing in obstructive respiratory diseases such as COPD have been suggested to increase fatty acid consumption^21^, with previous urine metabolomic studies supporting this finding with increased products of fatty acid catabolism^10^. Previous metabolomic studies of airway samples in preterm infants who later developed BPD have also shown decreased quantities of metabolites involved in β-oxidation of fatty acids^25^, as well as increases in acylcarnitines, which are released following β-oxidation of fatty acids during oxidative stress^26^. Similarly, altered β-oxidation of fatty acids^22^ and increases in serum acylcarnitine have also been noted in COPD^11,27^. We observed significantly decreased capric and caprylic acids in the POLD group. Capric and caprylic acids, both medium-chain fatty acids, have anti-inflammatory and antioxidant effects^28^ in porcine models of intestinal disease. Whether these metabolites have similar roles in the lung is speculative. We also saw a reduction of the very-long chain fatty acid ceratinic (also known as hexacosanoic) acid in the POLD group, likely related to increased consumption for energy release owing to inflammatory processes and oxidative stress. β-oxidation of very-long chain fatty acids in peroxisomes leads to the production of hydrogen peroxide (H_2_O_2_)^29^, a reactive oxygen species (ROS) resulting in oxidative damage and altered intracellular signaling. Increase in peroxisome activity, due to increased fatty acid metabolism, leads to peroxisome-induced oxidative stress^30^, with peroxisomal enzymes responsible for fatty acid breakdown and H_2_O_2_ production disproportionately upregulated compared to H_2_O_2_-scaveging enzymes, such as catalase, in rodent models^31^. Capric, caprylic and ceratinic acids had linear relationships with spirometry values across the preterm-born children, suggesting that β-oxidation of very-long chain fatty acids generally has an association with lung function.

Capric and caprylic acid, along with myristic acid were also implicated in fatty acid biosynthesis, another significantly altered process in the POLD group when compared with control groups. Fatty acid metabolism impairments have been observed in airway secretions from patients with COPD both during the stable phase and during acute exacerbations^32^. Macrophage activity activates and regulates COPD-related pulmonary inflammation^33^, and fatty acid metabolism is intrinsically linked with metabolic reprogramming of macrophages. Fatty acid biosynthesis has been shown to enhance pro-inflammatory activity and interleukin synthesis by macrophages, whereas fatty acid oxidation has a role in anti-inflammatory macrophage activity^34^. Our previous urine proteome study of PLD also suggested increased macrophage activity in POLD^14^.

We observed reduced levels of key metabolites related to glutathione synthesis and recycling in our POLD group. Glutathione provides potent defense against pulmonary oxidative injury, with studies of healthy adults demonstrating higher glutathione levels in the airways than in serum^35^. Animal models demonstrate pulmonary glutathione depletion enhances oxygen toxicity^36^. One pathway of glutathione consumption is in the removal of H_2_O_2_by conversion of reduced glutathione to glutathione disulfide, catalysed by the peroxisomal enzyme glutathione peroxidase^30^. Although not identical to our findings, decreased quantities of metabolites involved with glutathione metabolism, and thereby increased oxidative stress, have been observed in other respiratory pathologies, including COPD. Identical metabolites are often not found in such studies but given the different populations (e.g. adults vs. children), different samples analysed (e.g. blood, urine, EBC, BAL) and even different methodologies for acquiring similar samples, identical metabolites are often not noted between studies^37^, but importantly similar overall metabolic process, in this case metabolites involved in oxidant/antioxidant processes, are reported. Decreased alanine, pyroglutamic acid, glutamic acid and glutathione have been reported in a metabolomic study of murine lungs and in bronchoalveolar lavage fluid from adults with pulmonary inflammation and respiratory failure^38,39^. Pyroglutamic acid, glutamic acid, alanine and glutathione levels are decreased in targeted assay and/or metabolomic studies of serum from adults with COPD^22,23^, with pyroglutamic acid quantity being associated with a pulmonary emphysema phenotype^22^. We recently described reduced pyroglutamic acid in the airway metabolome for preterm-born school-aged children with BPD^40^. Whilst we did not detect glutathione in either its reduced or oxidized form, glutathione has a short half-life of approximately 10 min^41^, thus making its detection in urine challenging.

In contrast to the several altered metabolic pathways affected in the POLD group, suggestive of an ongoing active disease process, we observed far fewer significantly altered metabolites within the pPRISm group compared to the two control groups. This suggests pulmonary structural abnormalities in this group, rather than an active disease process, may be responsible for the lung function deficits observed in pPRISm. Only one metabolic process, namely tryptophan metabolism, was altered in the pPRISm group when compared to the Term control group. Tryptophan is an essential amino acid, and deficiency limits protein synthesis, causing cellular dysfunction and decreased proliferation. Reduced plasma tryptophan levels have been observed in COPD, particularly during acute exacerbations^42^. Reduced tryptophan metabolism, as suggested by our results, can also lead to reduced production of kynurenine. Kynurenine promotes naïve CD4+ T-cells to become anti-inflammatory T-regulator lymphocytes, rather than highly-inflammatory Th17 lymphocytes^43^. Our previous analysis of the urine proteome in PLD suggested increased inflammation and altered T-lymphocyte biology in the pPRISm group^14^. Additional work will be required to confirm these observations in other cohorts of preterm-born children and adults.

This exploratory study represents the first time, to our knowledge, that the urinary metabolome of PLD has been studied in childhood. Our study has been performed in one of the largest contemporary preterm-born paediatric populations available, who would have experienced modern standards of neonatal care. Composition of the urinary metabolome can be affected by dietary intake^44^, for which we had insufficient information to adjust for in our analyses. Future and replication studies should also consider the effect of participants dietary intake on the urinary metabolome. We withheld respiratory medications for the recommended period of time prior to spirometry, as per guidelines^16^, and whilst some inhaled medications, particularly corticosteroids, may have a more prolonged effect on the metabolome, we did not see any significant relationships with respiratory medications in our multivariable regression models. We adjusted our metabolite concentrations for dilutional effects using urinary creatinine, which is a widely accepted and recommended practice in urine metabolomic studies^20^. However, as our samples were collected at the time of spirometry, they were not necessarily early morning specimens, nor 24-h urine collections, which may reveal greater metabolomic differences. Our cohort was predominantly ethnically white, and future studies should aim to assess the metabolome for preterm-born individuals from other ethnic backgrounds. A robust method for metabolite annotation was employed, ensuring accuracy of metabolite identification; however, in relation to the whole known human metabolome, a relatively limited number of metabolites were successfully annotated. A minority of significantly altered metabolites were successfully mapped to metabolic pathways in the POLD group, however this is not an uncommon outcome of enrichment analysis methods. Our results require replication in a validation cohort, but we were limited by a lack of similar contemporaneous cohorts to study.

In conclusion, we have demonstrated active metabolic processes with multiple significantly altered metabolites in the urinary metabolome of children with a POLD phenotype, including changes in β-oxidation of very-long chain fatty acids, fatty acid biosynthesis and glutathione metabolism. These changes imply increased cellular energy requirements and oxidative stress which have also been observed in COPD. In contrast, the metabolome appears more stable in pPRISm with a suggestion of altered tryptophan metabolism. Whether this phenotype is associated more with structural abnormalities rather than metabolic ones is speculative and will require further study.

## Electronic supplementary material

Below is the link to the electronic supplementary material.

Supplementary Material 1

## Data Availability

The data generated and analysed that support the findings of this study are included in this published article [and its supplementary information file, Supplementary File.pdf]. Further data from the RHiNO study are available to research collaborators subject to confidentiality and non-disclosure agreements. Contact Professor Sailesh Kotecha (kotechas@cardiff.ac.uk) for any data requests.
