# Supplementary material for: Similarities of metabolomic disturbances in prematurity-associated obstructive lung disease to chronic obstructive pulmonary disease
Source: Sci Rep. 2024 Oct 7;14:23294. doi: 10.1038/s41598-024-73704-1 (PMC11458810; doi:10.1038/s41598-024-73704-1)
Supplement: Supplementary file 1 — Supplementary Material 1 [file 41598_2024_73704_MOESM1_ESM.pdf]

Similarities of metabolomic disturbances in prematurity-associated obstructive lung disease to chronic obstructive pulmonary disease – Supplementary Methods, Tables and Figures

Christopher W Course MBBCh[1], Philip A Lewis PhD[2], Sarah J Kotecha PhD[1], Michael Cousins PhD[1,3], Kylie Hart PhD[3], Kate J Heesom PhD[2], W John Watkins PhD[1], Sailesh Kotecha FRCPCH, PhD[1].

[1]Department of Child Health, School of Medicine, Cardiff University, Cardiff, United Kingdom.

[2]Faculty of Life Sciences, University of Bristol, Bristol, United Kingdom

[3]Department of Paediatrics, Cardiff and Vale University Health Board, Cardiff, United Kingdom

Corresponding Author: Professor Sailesh Kotecha

Department of Child Health

Cardiff University School of Medicine

Heath Park

Cardiff CF14 4XN

United Kingdom

kotechas@cardiff.ac.uk

Supplementary Methods: Description of the analysis protocol used at West Coast Metabolomics Centre:

To extract the metabolite content of the urine sample, 30µL of urine was mixed with 1ml of extraction solution (composed of acetonitrile, isopropranolol and water in a 3:3:2 (v/v/v) ratio) on ice. Samples were vortexed for 10 minutes, agitated for 5 min at 4°C on an orbital mixing chilling/heating plate before centrifugation for 2 minutes at 14,000 rcf. The supernatant was divided into two 450µL aliquots, with one aliquot kept in reserve. 100µL was removed from the remaining aliquot for sample analysis with another 100µL removed for use in the pool sample. All processed samples were stored at -20°C pending analysis.

100µL of each sample was fractionated using an Agilent 6890 gas chromatograph (Agilent, Santa Clara, CA, USA), controlled using Leco ChromaTOF software v2.32 (LECO, St. Joseph, MI, USA), in a Rtx-5Sil MS column (Restek, Bellafonte, PA, USA) (30m length x 0.25mm internal diameter with 0.25µm film made of 95% dimethyl/5%diphenylpolysiloxane). Quality control (QC) samples comprised two method blanks (involving all the reagents and equipment used to control for laboratory contamination) and four calibration curve samples, which spanned one order of dynamic range and consisted of 31 pure reference compounds. Column temperature was maintained between 50-330°C, with a helium mobile phase. Injection volumes of 0.5µL were used, with injection temperatures starting at 50°C, increased to a maximum temperature of 250°C by 12°Cs^{-1}. Oven temperature program was set to 50°C for 1 min, then increased at 20°C min^{-1} to 330°C, and held constant for 5 min. The analytical GC column was protected by a 10m long empty guard column which was cut by 20cm intervals whenever the reference mixture QC samples indicated problems caused by column contaminations. This sequence of column cuts has been validated, with no detrimental effects being detected with respect to peak shapes, absolute or relative metabolite retention times or reproducibility of quantifications. This chromatography method yields optimal retention and separation of primary metabolite classes (amino acids, hydroxyl acids, carbohydrates, sugar acids, sterols, aromatics, nucleosides, amines and miscellaneous compounds) with narrow peak widths of 2–3s and very good within-series retention

time reproducibility of better than 0.2s absolute deviation of retention times. Automatic liner exchanges after each set of 10 injections were used, which reduces sample carryover for highly lipophilic compounds.

All spectra were acquired using a Leco Pegasus IV (LECO, St. Joseph, MI, USA) time-of-flight mass spectrometer, with unit mass resolution at 17 spectra s^{-1} from 80-500Da at -70eV ionization energy and 1800V detector voltage with a 230°C transfer line and a 250°C electron ion source. Raw data files were normalised to QC/pool samples using the systematic error removal by random forest (SERRF) method(1). Raw data files were processed and metabolites identified with the BinBase metabolomics database(2), using an algorithm based on the following: validity of chromatogram (<10 peaks with intensity>10^7 counts s^{-1}), unbiased retention index marker detection (MS similarity >800, validity of intensity range for high m/z marker ions), retention index calculation by 5th order polynomial regression. Spectra were cut to 5% base peak abundance and matched to database entries from most to least abundant spectra using the following matching filters: retention index window ±2,000 units (equivalent to about ±2s retention time), validation of unique ions and apex masses (unique ion must be included in apexing masses and present at >3% of base peak abundance), mass spectrum similarity must fit criteria dependent on peak purity and signal/noise ratios and a final isomer filter. Quantification of metabolites were reported as spectral peak height of the unique ion detected (m/z value) at the specific retention index. Peak heights were more precise for metabolites with low abundance than peak areas, due to the larger influence of baseline determinations on areas compared to peak heights. Raw data files were processed, and metabolites annotated using the BinBase database(2) with a standardised algorithm.

References for additional methods:

1. Fan S, Kind T, Cajka T, Hazen SL, Tang WHW, Kaddurah-Daouk R, et al. Systematic Error Removal Using Random Forest for Normalizing Large-Scale Untargeted Lipidomics Data. Anal Chem. 2019;91(5):3590-6.

2. Lai Z, Tsugawa H, Wohlgemuth G, Mehta S, Mueller M, Zheng Y, et al. Identifying metabolites by integrating metabolome databases with mass spectrometry cheminformatics. Nat Methods. 2018;15(1):53-6.

Additional File Table 1: All identified detected metabolites, and the number and percentage of samples in which they were detected.

Metabolite	Retention Index	m/z	PubChem ID	No. of samples	Percentage of samples
1,2,4-benzenetriol	521803	239	10787	291	100
1,2-anhydromyoinositol	651472	318	119054	291	100
1-hexadecanol	679596	299	2682	287	98.6
1-kestose	1123027	361	440080	256	88.0
1-methyladenosine	829921	259	27476	287	98.6
1-methylinosine	829921	259	27476	201	69.1
1-monostearin	959214	203	24699	291	100
2,3-dihydroxybutanoic acid	384796	292	250402	291	100
2,8-dihydroxyquinoline	626989	290	97250	291	100
2-aminophenol	438445	150	NA	282	96.9
2-deoxytetronic acid	433456	189	150929	291	100
2-hydroxy-2-methylbutanoic acid	264833	145	95433	290	99.7
2-hydroxyglutaric acid	506306	247	43	291	100
2-hydroxyhippuric acid	725465	206	10253	291	100
2-hydroxypyrazinyl-2-propenoicacidethylester	493127	121	5371086	291	100
2-hydroxyvaleric acid	309587	131	98009	291	100
2-isopropylmalic acid	508690	275	5280523	203	69.8
2-ketoisocaproic acid	290473	89	70	291	100
2-methylglyceric acid	372491	219	560781	290	99.7
2-monopalmitin	890356	129	123409	291	100
2-picolinic acid	383668	180	1018	291	100
3,3-hydroxyphenyl-3-hydroxypropionic acid	632357	267	102959	291	100
3,3-hydroxyphenylpropionic acid	583925	192	91	290	99.7
3,4-dihydroxybenzoic acid	620200	193	72	291	100
3,4-dihydroxycinnamic acid	748847	219	689043	290	99.7
3,4-dihydroxyhydrocinnamic acid	673176	179	348154	291	100
3,4-dihydroxyphenylacetic acid	625046	179	547	291	100
3,6-anhydro-D-galactose	588886	231	16069996	291	100
3-aminoisobutyric acid	452655	248	64956	291	100
3-hydroxy-3,4-hydroxy-3-methoxyphenylpropionic acid	688753	297	NA	288	99.0
3-hydroxy-3-methylglutaric acid	521554	247	1662	291	100
3-hydroxyanthralinic acid	640146	354	NA	291	100
3-hydroxyphenylacetic acid	527648	164	12122	291	100
3-hydroxypropionic acid	269265	177	68152	291	100
3-phosphoglycerate	610734	227	724	282	96.9
4-hydroxybenzoate	537925	223	135	291	100
4-hydroxyhippuric acid	784581	294	151012	291	100
4-hydroxyphenylacetic acid	542795	179	127	291	100
4-methylcatechol	416586	268	9958	285	97.9
4-pyridoxic acid	673225	309	6723	290	99.7
5-aminovaleric acid	536657	174	138	291	100

5-deoxy-5-methylthioadenosine	967036	236	439176	291	100
5-hydroxy-3-indoleacetic acid	777606	290	1826	291	100
5-hydroxymethyl-2-furoic acid	497561	123	80642	290	99.7
6-deoxyglucitol	596111	319	151266	290	99.7
7-methylguanine	768706	294	11361	291	100
aconitic acid	586815	229	643757	291	100
adenine	646534	264	190	291	100
adenosine	918039	236	60961	291	100
adipic acid	474435	111	196	291	100
alanine	244189	116	5950	291	100
allantoic acid	726050	259	203	290	99.7
alloxanoic acid	785329	331	94146	189	64.9
alpha-ketoglutarate	507392	198	51	291	100
aminomalonate	455754	218	100714	291	100
anthranilic acid	530297	266	NA	287	98.6
arabitol	572730	103	94154	291	100
arachidic acid	856421	117	10467	291	100
ascorbic acid	672898	332	54670067	290	99.7
asparagine	553743	231	6267	291	100
aspartic acid	480387	232	5960	291	100
azelaic acid	610551	317	19347555	290	99.7
benzoic acid	339067	179	243	291	100
beta-alanine	435564	248	239	291	100
beta-gentiobiose	973116	204	441422	291	100
beta-mannosylglycerate	774364	204	5460194	231	79.4
biphenyl	426625	154	7095	285	97.9
butane-2,3-diol	205778	117	262	291	100
butyrolactam	277199	142	12025	291	100
capric acid	452386	229	2969	291	100
caprylic acid	343457	201	379	291	100
catechol	376695	254	289	291	100
cellobiose	932179	204	6255	291	100
ceratinic acid	1033286	145	10469	250	85.9
cholesterol	1078536	129	5997	290	99.7
citramalic acid	456203	247	1081	291	100
citric acid	617342	273	311	291	100
citrulline	621404	157	9750	291	100
conduritol-beta-epoxide	675635	318	9989541	220	75.6
creatinine	502599	115	588	291	100
cystathionine	772979	218	439258	239	82.1
cysteine	500158	220	5862	291	100
cysteine-glycine	715335	220	439498	289	99.3
cystine	804619	218	595	291	100
dehydroabietic acid	850374	239	94391	207	71.1
dehydroascorbic acid	633423	173	440667	291	100

deoxypentitol	528774	231	270738	291	100
digalacturonic acid	950338	233	439694	222	76.3
digitoxose	521798	117	94168	291	100
diglycerol	591074	103	42953	291	100
dihydro-3-coumaric acid	582960	192	91	291	100
docosenoic acid	911928	129	6433893	291	100
dodecanol	507619	243	8193	291	100
enolpyruvate	234394	217	1005	286	98.3
erythritol	471922	217	222285	291	100
erythronic acid lactone	407495	247	5325915	286	98.3
erythrose major	443306	205	439574	185	63.6
ethanolamine	342561	174	700	291	100
ferulic acid	732779	338	445858	273	93.8
fructose	639442	307	439709	291	100
fucose	578299	160	439650	291	100
fumaric acid	390016	245	444972	291	100
furoylglycine	553990	95	21863	291	100
galactinol	1015529	204	NA	291	100
galactitol	669079	319	5460044	291	100
galactonic acid	690882	292	128869	291	100
galactose	648756	319	439357	291	100
glucoheptulose	828606	217	5459879	290	99.7
gluconic acid	693148	333	6857417	291	100
gluconic acid lactone	645815	220	7027	291	100
glucose	659798	319	64689	291	100
glucuronic acid	665901	333	94715	291	100
glutamic acid	529100	246	33032	291	100
glutamine	600000	156	5961	291	100
glutaric acid	421596	261	743	291	100
glycerol	344466	205	753	291	100
glycerol-3-galactoside	805227	204	1604861<br>8	291	100
glycerol-alpha-phosphate	590747	357	754	291	100
glycine	368707	248	750	291	100
glycolic acid	227636	177	757	291	100
glycyl proline	691357	174	3013625	291	100
guanidinosuccinate	699521	444	NA	282	96.9
guanine	744307	352	764	287	98.6
heptadecanoic acid	751309	117	10465	291	100
hippuric acid	638579	206	NA	291	100
histidine	663790	154	6274	291	100
homocystine	874865	128	10010	289	99.3
homovanillic acid	601084	326	1738	291	100
hydroxycarbamate	325318	278	1663916<br>1	286	98.3
hydroxyproline dipeptide	879596	156	6115952<br>6	289	99.3

hypoxanthine	619128	265	790	291	100
indole-3-acetate	684929	202	802	291	100
indole-3-lactate	764586	202	92904	291	100
indoxyl sulfate	577333	277	10258	291	100
isocitric acid	617338	245	5318532	291	100
isoleucine	359251	158	6306	291	100
isomaltose	983199	160	439193	231	79.4
isopropylbenzene	240619	105	7406	291	100
isothreonic acid	489385	292	151152	291	100
itaconic acid	386511	147	811	291	100
kynurenic acid	726186	231	3845	291	100
kynurenine	769709	218	2524586<br>2	277	95.2
lactic acid	217657	191	612	291	100
lactose	935640	191	440995	283	97.3
lactulose	929908	204	11333	291	100
lauric acid	547906	117	3893	291	100
leucine	346357	158	6106	291	100
levoglucosan	569637	204	2724705	291	100
levoinositol	651238	432	NA	291	100
lysine	663483	317	5962	291	100
maleimide	245118	154	10935	291	100
malic acid	463180	233	525	290	99.7
maltose-1	946601	204	439186	291	100
mannose	645856	205	18950	291	100
metanephrine	621765	297	21100	291	100
methanolphosphate	289520	241	13130	162	55.7
methionine	483560	176	6137	290	99.7
methylmaleic acid	418804	259	643798	282	96.9
montanic acid	1087377	117	10470	250	85.9
myo-inositol	730022	305	892	291	100
myristic acid	634414	285	11005	291	100
N-acetylaspartic acid	548028	158	65065	291	100
N-acetylmannosamine	722897	319	439281	291	100
N-acetylputrescine	595523	174	122356	291	100
N-carbamoylaspartate	611345	257	93072	291	100
N-carbamylglutamate	651275	257	121396	291	100
n-epsilon-trimethyllysine	512366	118	440121	291	100
nicotinic acid	366992	180	938	290	99.7
N-methylglutamic acid	455629	98	439377	291	100
nonadecanoic acid	822782	117	12591	291	100
noradrenaline	754841	174	439260	291	100
octadecanol	755409	327	8221	291	100
oleamide	849710	144	5283387	211	72.5
oleic acid	781527	339	445639	271	93.1
ornithine	619196	142	8874724<br>8	291	100

orotic acid	586317	254	967	291	100
oxoproline	485935	156	7405	291	100
palatinitol	996670	204	88735	269	92.4
parabanic acid	464991	100	67126	288	99.0
p-cresol	280360	165	2879	291	100
pentitol	563801	307	827	291	100
pentose	540818	103	229	291	100
phenol	218927	151	996	291	100
phenylalanine	537804	192	6140	291	100
phosphate	361492	314	1004	289	99.3
pimelic acid	523205	155	385	291	100
pinitol	622466	260	164619	234	80.4
proline	364716	142	145742	291	100
pseudo uridine	813899	217	15047	291	100
psicose	635244	307	NA	291	100
p-tolyl glucuronide	847531	180	154035	291	100
putrescine	588119	174	1045	290	99.7
pyrogallol	495011	239	1057	291	100
pyrophosphate	327517	110	1023	291	100
pyruvic acid	213805	174	1060	291	100
quinic acid	634900	345	6508	291	100
quinolinic acid	581638	296	1066	290	99.7
raffinose	1120886	361	439242	230	79.0
ribitol	575497	217	827	291	100
ribonic acid	599680	292	5460677	291	100
ribose	553071	217	1097565<br>7	291	100
saccharic acid	699211	333	33037	291	100
salicylaldehyde	405583	193	6998	284	97.6
salicylic acid	480699	267	338	286	98.3
serine	395020	204	5951	291	100
serotonin	863824	174	5202	289	99.3
shikimic acid	611100	204	8742	291	100
sinapinic acid	788416	338	637775	150	51.5
sophorose	959716	319	NA	286	98.3
sorbitol	667922	217	5780	291	100
succinic acid	370608	247	1110	291	100
sucrose	915139	271	5988	291	100
tagatose	631835	307	439312	291	100
tartaric acid	534291	292	444305	289	99.3
threitol	467595	217	169019	291	100
threonic acid	497572	292	5460407	291	100
threonine	409568	218	6288	291	100
thymine	420133	255	1135	291	100
trehalose	948197	191	7427	290	99.7
triethanolamine	531892	262	7618	202	69.4

tryptophan	774603	130	6305	291	100
tyrosine	671252	218	6057	291	100
UDP-glucuronic acid	585473	217	17473	291	100
uracil	385735	241	1174	291	100
urea	323728	189	1176	291	100
uric acid	730691	441	1175	291	100
uridine	861508	217	6029	263	90.4
urocanic acid	699866	267	736715	291	100
valine	313502	144	6287	291	100
vanillic acid	597845	297	8468	291	100
xanthine	701688	353	1188	291	100
xanthosine	926133	325	64959	258	88.7
xanthurenic acid	795062	406	5699	290	99.7
xylitol	567437	217	6912	291	100
xylonic acid	589278	333	6602431	290	99.7
xylonic acid isomer	590775	189	10264	290	99.7
xylose	544100	103	135191	291	100
xylulose	553450	173	439205	291	100

Supplementary File Figure 1: Log_{10} urinary creatinine values for the four study groups

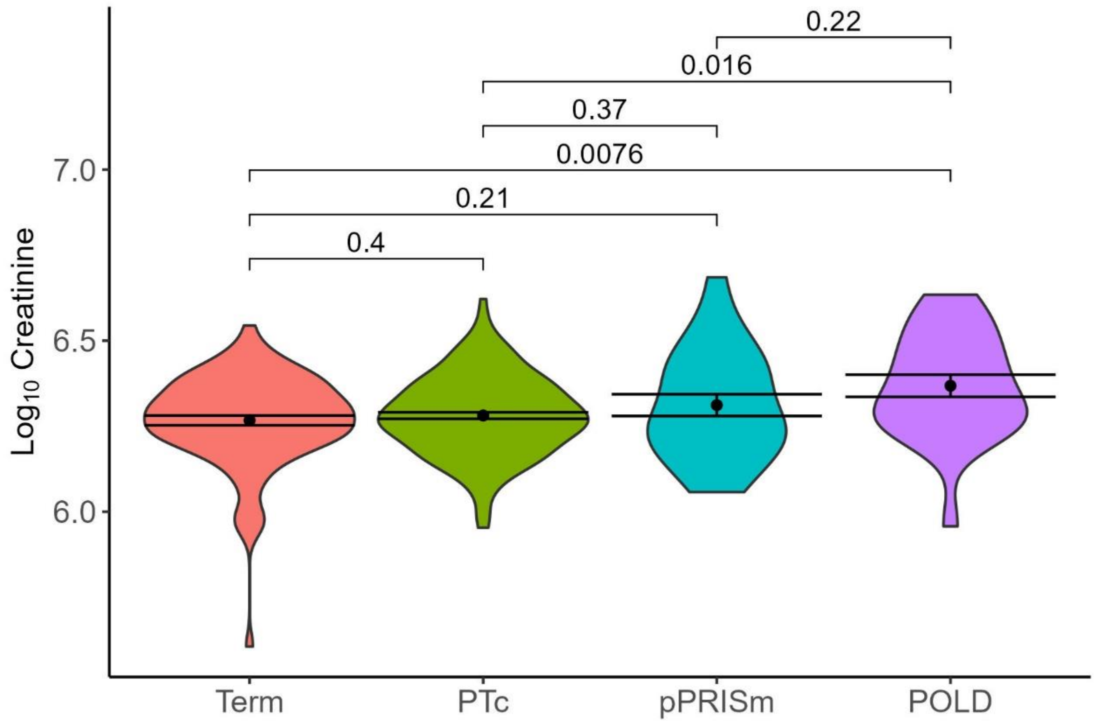

Violin Plots of urine creatinine values for the four study groups. Black dot and bars show mean and standard error of the mean (SEM). Bars give p-values from ANOVA with post-hoc Bonferroni correction for between group comparisons. PTc: Preterm-born controls. pPRISm: Prematurity-associated Preserved Ration Impaired Spirometry. POLD: Prematurity-associated obstructive lung disease.

Supplementary File Figure 2: Diagram representing glutathione metabolism, highlighting altered metabolites within the POLD group and links with abnormalities in COPD described in the literature.

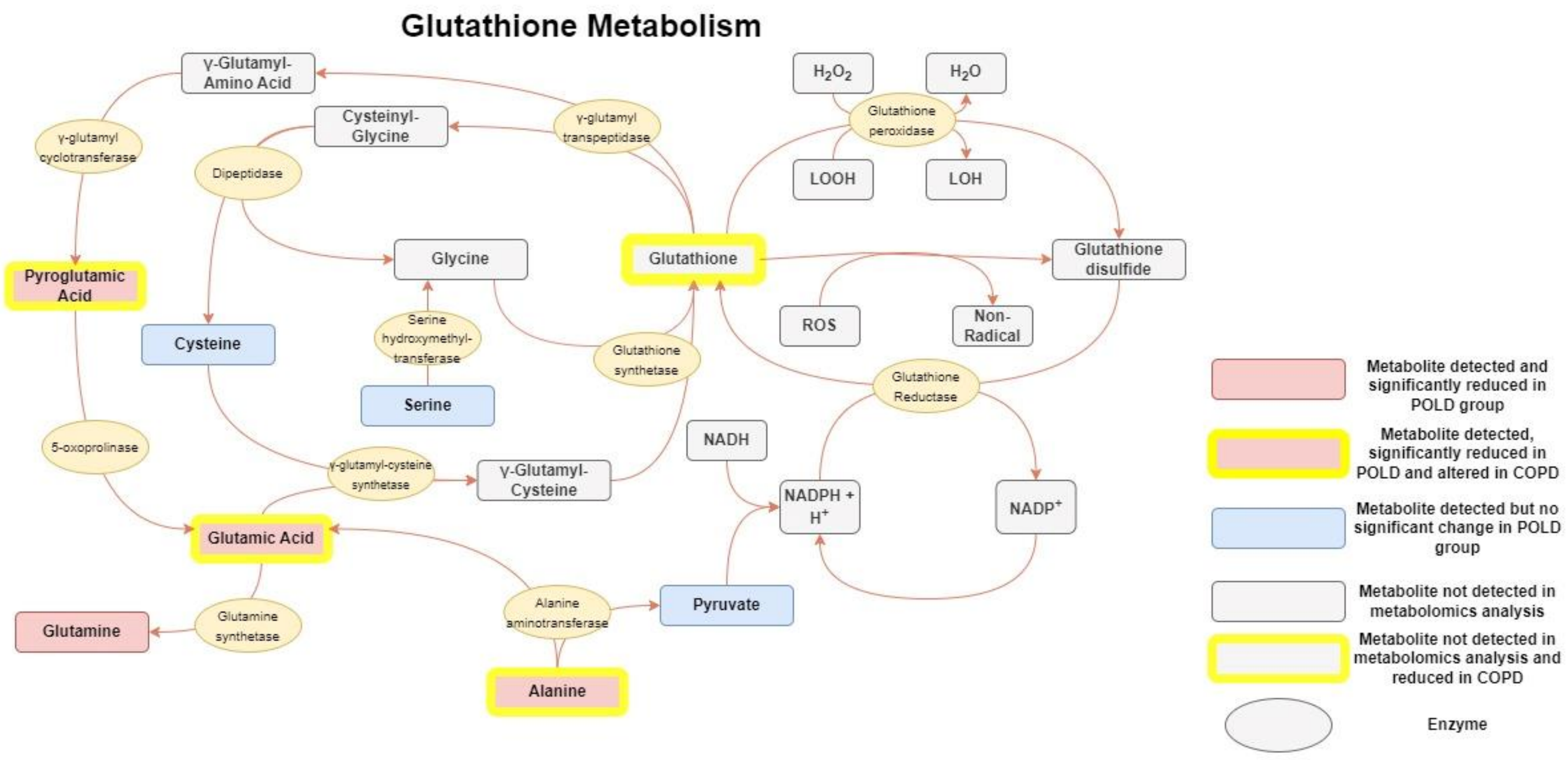

Additional File Table 2: Significantly altered metabolites between POLD and pPRISm groups compared with term-born controls.

Metabolite	Retention Index	m/z	PubChem ID	% of samples	Log_2FC	p-value
**POLD vs Term n=23 v 94**						
biphenyl	426625	154	7095	98.3	-0.42	0.0003
xylonic acid	589278	333	6602431	99.1	-0.94	0.0003
myristic acid	634414	285	11005	100	-0.48	0.0005
guanine	744307	352	764	99.1	-0.48	0.0007
threitol	467595	217	169019	100	-0.73	0.002
xylose	544100	103	135191	100	-1.14	0.002
enolpyruvate	234394	217	1005	100	-0.43	0.002
2-ketoisocaproic acid	290473	89	70	100	-0.50	0.002
glutamic acid	529100	246	33032	100	-0.41	0.002
pentose	540818	103	229	100	-1.51	0.002
butane-2,3-diol	205778	117	262	100	-0.81	0.003
2-hydroxyhippuric acid	725465	206	10253	100	-0.71	0.003
5-hydroxymethyl-2-furoic acid	497561	123	80642	100	-1.32	0.003
furoylglycine	553990	95	21863	100	-0.63	0.004
urea	323728	189	1176	100	-1.55	0.004
ribitol	575497	217	827	100	-0.34	0.005
allantoic acid	726050	259	203	100	-0.48	0.005
galactinol	1015529	204	NA	100	-0.48	0.005
citramalic acid	456203	247	1081	100	-0.63	0.005
6-deoxyglucitol	596111	319	151266	99.1	-2.15	0.005
1-monostearin	959214	203	24699	100	-0.43	0.005
quinic acid	634900	345	6508	100	-2.82	0.005
capric acid	452386	229	2969	100	-0.32	0.006
caprylic acid	343457	201	379	100	-0.19	0.006
4-pyridoxic acid	673225	309	6723	100	-0.29	0.008
7-methylguanine	768706	294	11361	100	-0.29	0.008
gluconic acid	693148	333	6857417	100	-0.33	0.009
hypoxanthine	619128	265	790	100	-0.55	0.010
tartaric acid	534291	292	444305	99.1	-4.82	0.010
2-hydroxypyrazinyl-2-propenoicacidethylester	493127	121	5371086	100	-0.42	0.011
erythritol	471922	217	222285	100	-0.34	0.011
mannose	645856	205	18950	100	-0.63	0.011
deoxypentitol	528774	231	270738	100	-0.44	0.012
ribose	553071	217	10975657	100	-0.29	0.013
uric acid	730691	441	1175	100	-0.29	0.013
N-acetylmannosamine	722897	319	439281	100	-0.47	0.014
threonic acid	497572	292	5460407	100	-0.44	0.014
azelaic acid	610551	317	19347555	99.1	0.10	0.015
hippuric acid	638579	206	NA	100	-0.39	0.015
gluconic acid lactone	645815	220	7027	100	-0.26	0.018
butyrolactam	277199	142	12025	100	-0.28	0.018
adenine	646534	264	190	100	-0.24	0.019
shikimic acid	611100	204	8742	100	-0.70	0.021
pyrophosphate	327517	110	1023	100	-0.51	0.021
pyroglutamic acid	485935	156	7405	100	-0.28	0.023
N-carbamoylaspartate	611345	257	93072	100	-0.40	0.023
1-methylinosine	1026110	259	65095	68.4	-1.17	0.023
parabanic acid	464991	100	67126	100	-0.29	0.024
UDP-glucuronic acid	585473	217	17473	100	-0.56	0.025
indole-3-lactate	764586	202	92904	100	-0.37	0.025
maleimide	245118	154	10935	100	-0.28	0.027
thymine	420133	255	1135	100	-0.33	0.030
aconitic acid	586815	229	643757	100	-0.24	0.031

kynurenic acid	726186	231	3845	100	-0.44	0.034
uracil	385735	241	1174	100	-0.43	0.034
pyrogallol	495011	239	1057	100	-0.54	0.034
xanthine	701688	353	1188	100	-0.28	0.035
raffinose	1120886	361	439242	100	-0.49	0.036
cholesterol	1078536	129	5997	100	-0.64	0.036
glycerol	344466	205	753	100	-0.43	0.038
3-hydroxyanthralinic acid	640146	354	NA	100	-0.34	0.040
2-picolinic acid	383668	180	1018	100	-0.40	0.041
lactic acid	217657	191	612	100	-0.82	0.041
methanolphosphate	289520	241	13130	61.5	-1.40	0.041
benzoic acid	339067	179	243	100	-0.33	0.042
N-acetylaspartic acid	548028	158	65065	100	-0.24	0.042
cellobiose	932179	204	6255	100	-0.55	0.046
4-hydroxyphenylacetic acid	542795	179	127	100	-0.32	0.046
asparagine	553743	231	6267	100	-0.22	0.049
**pPRISm vs Term n = 25 v 94**						
oleic acid	781527	339	445639	100	-0.79	0.002
beta-mannosyl glycerate	774364	204	5460194	100	0.39	0.009
lactic acid	217657	191	612	100	-1.05	0.012
furoylglycine	553990	95	21863	100	-0.98	0.013
methanolphosphate	289520	241	13130	99.2	-0.73	0.019
indole-3-lactate	764586	202	92904	98.3	-0.48	0.028
glycyl proline	691357	174	3013625	99.2	0.44	0.028
butane-2,3-diol	205778	117	262	100	-0.48	0.034
3-hydroxyanthralinic acid	640146	354	NA	100	-0.30	0.044
biphenyl	426625	154	7095	100	-0.16	0.044
anthranilic acid	530297	266	NA	100	-0.31	0.044
3-(3-hydroxyphenyl)propionic acid	583925	192	91	98.3	0.44	0.046
xylonic acid	589278	333	6602431	100	-0.47	0.047

Additional File Table 3: Univariable linear regression analyses of identified metabolites of interest identified as part of Aspartate Metabolism and Urea Cycle with early and current life factors in preterm-born children.

Variable	Beta-Alanine			Fumaric Acid			Glutamine		
	**Beta**	**SE**	**p-value**	**Beta**	**SE**	**p-value**	**Beta**	**SE**	**p-value**
**Univariable Models**									
Sex, ref=Male	0.01	0.05	0.86	-0.02	0.02	0.30	-0.03	.02	0.16
Age at testing, years	-0.01	0.02	0.55	-0.004	0.01	0.64	0.002	0.01	0.79
Weight, z-score	-0.05	0.02	**0.021***	-0.02	0.01	*0.08*	-0.01	0.01	0.14
BMI, z-score	-0.05	0.02	**0.003***	-0.02	0.008	**0.049***	-0.01	0.01	*0.052*
Gestational age, weeks	<0.001	0.01	0.99	<0.001	0.004	0.95	0.003	0.004	0.47
Birthweight, z-score	-0.04	0.02	**0.032***	-0.01	0.01	0.19	-0.01	0.01	0.29
IUGR, ref=No IUGR	0.11	0.06	*0.098*	-0.005	0.03	0.88	-0.02	0.03	0.45
BPD, ref=No BPD	0.05	0.05	0.34	0.03	0.03	0.24	0.01	0.02	0.64
POLD, ref=PT_c	0.14	0.07	**0.043***	-0.07	0.03	*0.055*	-0.07	0.03	**0.034***
pPRISm, ref=PT_c	0.09	0.07	0.19	0.01	0.03	0.81	-0.02	0.03	0.61
Asthma, ref=No	0.01	0.06	0.90	-0.04	0.03	0.20	-0.04	0.03	0.12
**Multivariable Models**									
BMI, z-score	-0.04	0.02	**0.025***	-0.02	0.01	**0.021***	-0.02	0.01	**0.017***
Birthweight, z-score	-0.02	0.02	0.21	-	-	-	-	-	-
POLD, ref=PT_c	0.10	0.07	0.16	-0.08	0.03	**0.021***	-0.08	0.03	**0.012***

***and bold** indicates $p<0.05$, *italics* indicate $p<0.1$. Dashes indicate a variable where $p\geq0.1$ in univariable analysis and therefore not included in multivariable model. Variables with a p-value <0.1 in univariable model included in multivariable model. BMI: Body Mass Index, IUGR: Intrauterine growth restriction, BPD: Bronchopulmonary dysplasia, POLD: prematurity-associated obstructive lung disease, pPRISm: prematurity-associated preserved ratio impaired spirometry
